# Phosphorylation of the overlooked tyrosine 310 regulates the structure, aggregation, and microtubule- and lipid-binding properties of Tau

**DOI:** 10.1074/jbc.RA119.012517

**Published:** 2020-04-27

**Authors:** Nadine Ait-Bouziad, Anass Chiki, Galina Limorenko, Shifeng Xiao, David Eliezer, Hilal A. Lashuel

**Affiliations:** 1 Laboratory of Molecular and Chemical Biology of Neurodegeneration, Brain Mind Institute, École Polytechnique Fédérale de Lausanne (EPFL), CH-1015 Lausanne, Switzerland; 2 Shenzhen Key Laboratory of Marine Biotechnology and Ecology, College of Life Sciences and Oceanography, Shenzhen University, Shenzhen, China; 3 Department of Biochemistry and Program in Structural Biology, Weill Cornell Medical College, New York, New York

**Keywords:** Tau protein (Tau), tauopathy, neurodegenerative disease, phosphotyrosine, aggregation, microtubule, post-translational modification (PTM), phosphorylation, amyloid, lipid binding

## Abstract

The microtubule-associated protein Tau is implicated in the pathogenesis of several neurodegenerative disorders, including Alzheimer's disease. Increasing evidence suggests that post-translational modifications play critical roles in regulating Tau's normal functions and its pathogenic properties in tauopathies. Very little is known about how phosphorylation of tyrosine residues influences the structure, aggregation, and microtubule- and lipid-binding properties of Tau. Here, we sought to determine the relative contributions of phosphorylation of one or several of the five tyrosine residues in Tau (Tyr-18, -29, -197, -310, and -394) to the regulation of its biophysical, aggregation, and functional properties. We used a combination of site-specific mutagenesis and *in vitro* phosphorylation by c-Abl kinase to generate Tau species phosphorylated at all five tyrosine residues, all tyrosine residues except Tyr-310 or Tyr-394 (pTau-Y310F and pTau-Y394F, respectively) and Tau phosphorylated only at Tyr-310 or Tyr-394 (4F/pTyr-310 or 4F/pTyr-394). We observed that phosphorylation of all five tyrosine residues, multiple N-terminal tyrosine residues (Tyr-18, -29, and -197), or specific phosphorylation only at residue Tyr-310 abolishes Tau aggregation and inhibits its microtubule- and lipid-binding properties. NMR experiments indicated that these effects are mediated by a local decrease in β-sheet propensity of Tau's PHF6 domain. Our findings underscore Tyr-310 phosphorylation has a unique role in the regulation of Tau aggregation, microtubule, and lipid interactions. These results also highlight the importance of conducting further studies to elucidate the role of Tyr-310 in the regulation of Tau's normal functions and pathogenic properties.

## Introduction

The misfolding and aggregation of the microtubule (MT)-associated protein Tau have been linked to the pathogenesis of several neurodegenerative disorders (1), including Alzheimer disease (AD) (2), corticobasal degeneration (CBD) (3), progressive supranuclear palsy (3, 4), Pick's disease, argyrophilic grain disease (5), chronic traumatic encephalopathy (CTE) (6), and frontotemporal dementia with parkinsonism linked to chromosome 17 (FTDP-17) (7). In the brain of individuals affected by AD, aggregated and abnormally hyper-phosphorylated and modified forms of Tau, such as paired helical filaments (PHFs) and straight filaments (SFs), are found as the main component of the neurofibrillary tangles (NFTs), one of the key diagnostic pathological hallmarks of AD, in addition to amyloid plaques (8).

Tau is extensively post-translationally modified with phosphorylation being one of the predominant modifications (9). Although many phosphorylation sites of Tau identified in NFTs are also found in healthy neurons, at advanced disease stages Tau aberrant hyper-phosphorylation (10) encompasses more than 39 potential sites (11, 12), leading to a total phosphorylation level of Tau that is three to four times higher than that of normal Tau (13, 14). The presence of hyper-phosphorylated Tau in both soluble cytosolic forms and highly aggregated and insoluble forms within NFTs (13, 15) suggests that the pattern of hyper-phosphorylation or site-specific phosphorylation may play key roles in regulating its normal function(s) and/or pathogenic properties.

**Figure 1.**
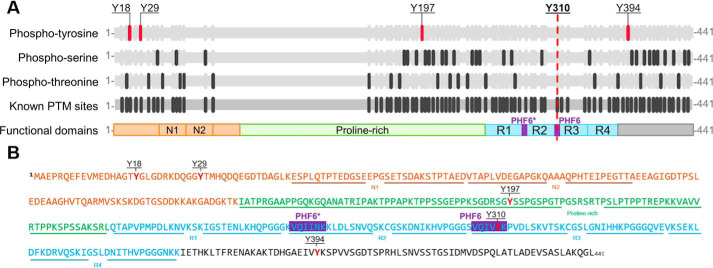
**Schematic depiction of the sequence and different domains in full-length Tau-441.** *A,* map and domains of full-length Tau with known post-translational modification sites (*gray*); phosphotyrosines (*magenta*), phosphoserines (*dark gray*), and phosphothreonines (*dark gray*). Furthermore, Tau contains different functional domains: N-terminal sites (*N1*, aa 45–74; *N2*, aa 75–103), a proline-rich region (aa 151–224), and four repeat domains (*R1*, aa 244–274; *R2*, aa 275–305; *R3*, aa 306–336; *R4*, aa 337–368). The microtubule-binding region contains 2 hexapeptide domains PHF6* (aa 275–280, *purple*) and PHF6 (aa 306–311, *purple*). Adapted from Ref. 78). *B,* the annotated sequence of full-length Tau. Tau possesses 5 tyrosine residues, as indicated in *red*, at positions Tyr-18, Tyr-29, Tyr-197, Tyr-310, and Tyr-394. Tyr-18 and Tyr-29 are localized in the N-terminal region of Tau (*orange*, N-terminal repeats N1 and N2 *underlined*), Tyr-197 in the proline-rich domain (*green*), Tyr-310 in the third repeat of the MTBR of Tau (*blue*) and Tyr-394 in the C-terminal part of the protein (*black*). PHF6 and PHF6* hexapeptides are indicated in *purple*.

Serine and threonine residues greatly outnumber tyrosine residues in the Tau sequence, which explains why phosphorylation at these residues has received predominant attention. Tau contains five tyrosine residues (Fig. 1), located at positions Tyr-18, Tyr-29, Tyr-197, Tyr-310 and Tyr-394, which are all susceptible to phosphorylation. Tau phosphorylation on tyrosine residues has been described only recently and remains understudied. A few tyrosine kinases have been shown to phosphorylate tyrosine residues in Tau both *in vitro*, in cell culture and *in vivo*. For example, Fyn (16, 17) and Lck (17), members of the Src kinase family, phosphorylate Tau on all five tyrosine residues, however, somewhat preferentially on residue Tyr-18; whereas members of the c-Abl kinase family (c-Abl and Arg) were reported to phosphorylate Tau on Tyr-394 (18, 19) and to a lesser extent on Tyr-197 and Tyr-310. Other non-SH3–containing tyrosine kinases have also been reported to phosphorylate Tau on residue Tyr-18, such as Syk (20) and Pyk2, the focal adhesion kinase (21); and on residue Tyr-197 by Tau tubulin kinase 1 (22). Additionally, it was shown that an increase in Tau tyrosine phosphorylation correlated with the formation of Tau aggregates (23). Furthermore, several reports have established that Abelson tyrosine kinase (c-Abl) becomes active in a variety of neurodegenerative diseases, including Parkinson's disease and several tauopathies (24–26), suggesting a likely role in the regulation of Tau tyrosine phosphorylation levels in disease.

Alternative splicing of exons 2, 3, and 10 of Tau produces 6 isoforms from chromosome 17. The splicing results in an assembly domain that contains three or four microtubule-binding repeats (27). The four repeats form the proteolysis-resistant core of Tau aggregates (28). PHF6* and PHF6 hexapeptides (Fig. 1, *A* and *B*) are important elements for Tau binding to microtubules and constitute the minimal motif allowing fibrillization (28, 30). K19 and K18 are fragments of Tau that comprise three and four repeat domains, respectively. These fragments have high aggregation and microtubule-binding propensities (31, 32). Whereas phosphorylation of serine and threonine residues exhibit a degree of clustering in the N- and C-terminal functional domains encompassing the repeat regions and MT-binding domains 1 and 2 (Fig. 1, *A* and *B*), there is only one tyrosine phosphorylation site (Tyr-310) within the MT-binding domain. This residue is located in the repeat peptide R3, which has also been shown to play key roles in regulating Tau aggregation, PHF formation, and membrane interactions (33, 34).

Interestingly, despite the prominent position of Tyr-310 within the aggregation-prone hexapeptide PHF6 (amino acids 306–311), only a handful of studies have specifically investigated the roles of this residue in regulating Tau structure, aggregation, and toxicity. Importantly, none of the previous studies (30, 35–42) specifically investigated the effects of Tyr-310 phosphorylation in the context of the full-length Tau protein, relying instead on the short PHF6-containing peptides, featuring PHF6-derived sequences as short as three amino acids (43). Santa-Maria *et al.* (44) showed that phosphorylation of Tyr-310 abolished the aggregation of the PHF6 fragment (amino acids 306–311) *in vitro*. Conversely, Inoue *et al.* (40, 45, 46) reported contradictory results, where phosphorylation of Tyr-310 resulted in a marked increase in the propensity of PHF6 peptide to form fibrils. Furthermore, whereas previous studies have suggested that Tau phosphorylation on tyrosine residues is an important modulator of Tau functions under both normal conditions and in the course of AD pathogenesis (16, 47), the role of each tyrosine residue in regulating Tau protein aggregation, MT-binding, and lipid-binding propensities has not been systematically investigated.

This knowledge gap combined with the location of Tyr-310 within the PHF6 domain of Tau prompted us to conduct more detailed investigations to: 1) determine the effects of tyrosine phosphorylation on the regulation of the normal function and pathogenic properties of full-length Tau; 2) elucidate the relative contribution of phosphorylation at each tyrosine residue; and 3) investigate the effect of phosphorylation at Tyr-310 on the structure, aggregation, and microtubule-binding of the full-length Tau and microtubule-binding domain-containing Tau K18 fragment. Toward achieving these goals, we utilized a combination of mutagenesis and *in vitro* phosphorylation using the c-Abl kinase to produce milligram quantities of Tau phosphorylated at single or multiple tyrosine residues. Our findings show that phosphorylation of Tyr-310 is sufficient to inhibit Tau aggregation and plays a key role in regulating Tau microtubule-binding properties and interactions with the membrane, thus underscoring the importance of further studies to elucidate the role of phosphorylation at this residue in regulating Tau function under normal and pathological conditions.

## Results

### c-Abl phosphorylates Tau on multiple tyrosine residues in vitro

**Figure 2.**
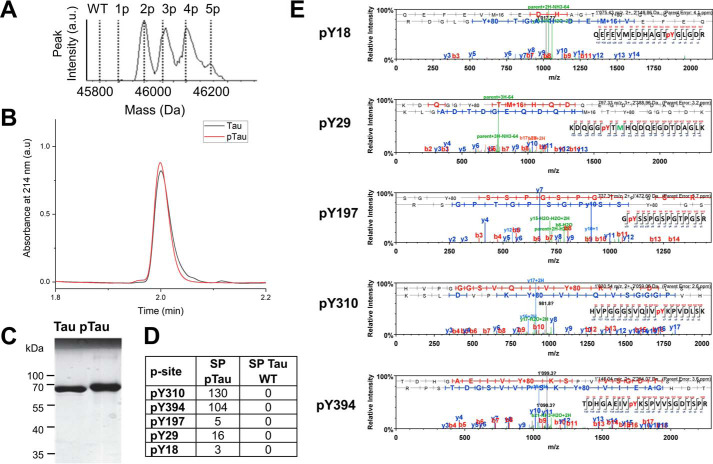
**c-Abl–mediated *in vitro* phosphorylation and characterization of Tau (*pTau*).** Phosphorylation was performed for 4 h, which leads to a mixture of Tau phosphorylated on two to five residues. Characterization of the RP-HPLC purified Tau and the pTau mixtures by LC-MS (*A*), UPLC (*B*), and SDS-PAGE (*C*). *D* and *E,* tandem LC/MS/MS of RP-HPLC purified pTau following trypsin digestion and peptides enrichment using a TiO_2_ resin. The analysis of the phosphopeptides was performed using the Scaffold version 4.0 (Proteome Software), and the number of phosphopeptides detected per phosphorylation site was reported as the spectral count (*SP*).

To investigate the role of tyrosine phosphorylation in regulating Tau structure, aggregation, and MT-binding properties, we first sought to develop conditions that allow for efficient phosphorylation of Tau *in vitro*. Toward this goal, we assessed the phosphorylation efficiency and specificity of c-Abl kinase for Tau. Recombinant full-length Tau was subjected to *in vitro* phosphorylation using recombinant c-Abl and the extent of phosphorylation and number of phosphorylation sites was systematically followed by LC/MS (Fig. 2 and Fig. S1). Fig. 2*A* shows that c-Abl phosphorylated Tau at multiple sites, up to 5 sites after 4 h of incubation in reaction buffer. Increasing the amount of kinase or incubation time did not cause a shift toward species with a higher number of phosphorylated tyrosine residues or relative phosphorylation levels (data not shown). Although RP-HPLC allowed the separation of the kinase from the phosphorylated Tau proteins, it was not possible to achieve separation of the different phosphorylated Tau species, which eluted in one peak as observed by the UPLC and a single band by SDS-PAGE (Fig. 2, *B* and *C*). To determine the exact tyrosine phosphorylation profile of Tau, the purified mixture of pTau was digested by trypsin and the resulting peptides were analyzed by LC/MS/MS. The analysis of the phosphorylated sites was performed using the Scaffold software (RRID:SCR_01435), which determines the abundance of phosphorylated peptides based on the spectral counting (SP). As shown in Fig. 2, *D* and *E*, c-Abl phosphorylated Tau on Tyr-18, Tyr-29, Tyr-197, Tyr-310 and Tyr-394, with Tyr-310 and Tyr-394 being the major phosphorylation sites.

### c-Abl phosphorylation of Tau significantly delays its aggregation

**Figure 3.**
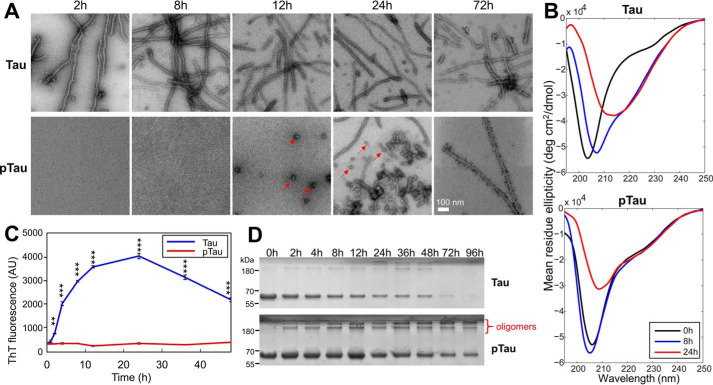
**Comparison of the aggregation properties of WT Tau and purified c-Abl–phosphorylated Tau (pTau).** 10 μm Tau and pTau were incubated for 48 h at 37 °C under shaking conditions, in the presence of 2.5 μm heparin and the extent of aggregation was monitored by EM (*scale bar* = 100 nm for all images) (*A*), CD spectroscopy (*B*), ThT fluorescence (*C*), and sedimentation assays (*D*). Quantification of the supernatant band intensity could not be performed due to the presence of soluble nonpelletable oligomeric species in the pTau sample. In *C,* two-way ANOVA with Tukey's multiple comparisons test, significance values are indicated by: **, *p* < 0.01; ***, *p* ≪ 0.001; time points at 0 and 1 h were not significantly different.

To determine the effect of tyrosine phosphorylation on Tau aggregation, we carried out *in vitro* phosphorylation of Tau using the c-Abl kinase and purified the mixture of Tau-phosphorylated proteins using RP-HPLC. Next, we compared *in vitro* aggregation of the nonphosphorylated to c-Abl–phosphorylated Tau (Fig. 3) by incubating and monitoring the aggregation of both proteins (at 10 μm) over 2 days at 37 °C under shaking conditions, in the presence of 2.5 μm heparin. We found that phosphorylated Tau (pTau) was unable to form canonical β-sheet–containing fibrils under conditions where the unmodified Tau readily forms fibrils, as demonstrated by: 1) EM (Fig. 3*A*); 2) the absence of conversion of Tau conformation to β-sheet structure as discerned by circular dichroism (CD) (Fig. 3*B*); and 3) the fact that the thioflavin T (ThT) fluorescence remained at baseline levels (Fig. 3*C*). However, large oligomers were discernible by EM at 12 h, and longer fibrils were apparent only after 24–48 h incubation. These observations were further confirmed by the fact that the sedimentation assay showed an increase of the SDS-resistant oligomeric species over time in the pTau sample, but not for the unmodified protein (Fig. 3, *A* and *D*).

It is likely that oligomeric species observed in the unmodified Tau sample were ON-pathway to fibril formation and represent intermediate species that disappeared by the end of the reaction through equilibrium shift toward fibrillar structures. In contrast, the oligomers formed by pTau might represent OFF-aggregation pathway species that did not proceed to form fibrils and remained in the sample at 96 h (Fig. 3*D*). These results demonstrate that phosphorylation of Tau at multiple tyrosine residues significantly delays its aggregation and fibril formation and results in the accumulation of phosphorylated Tau oligomers.

### c-Abl–mediated inhibitory effects on Tau aggregation depend on the site of tyrosine phosphorylation

**Figure 4.**
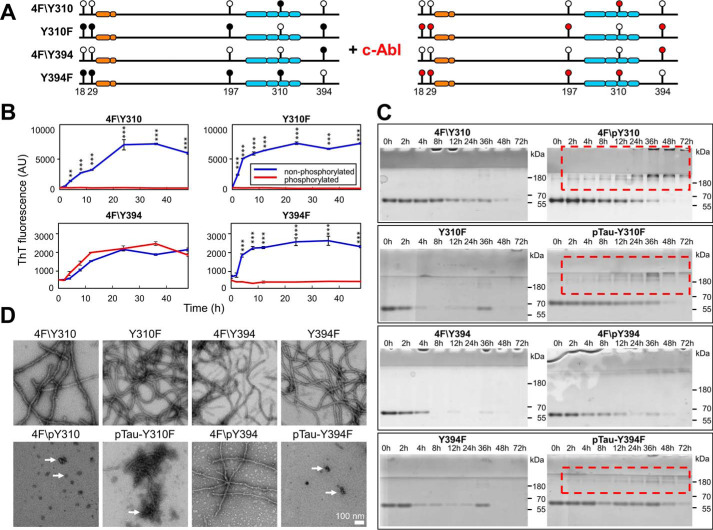
**Comparison of the aggregation behavior of nonphosphorylated and phosphorylated Tyr → Phe Tau mutants.** *A,* schematic depiction showing the position of Tau tyrosine residue Tyr → Phe mutations (*filled circle,* Tyr; *open circle*, Phe) and the tyrosine sites phosphorylated by c-Abl (*red circles*). For the aggregation studies, 10 μm non- and phosphorylated Tyr → Phe Tau mutants were incubated for 48 h at 37 °C under shaking conditions, in the presence of 2.5 μm heparin and the extent of aggregation was monitored by ThT fluorescence (*B*), sedimentation assays (*C*), and EM (*scale bar* = 100 nm for all images) (*D*). *B,* kinetics of aggregation of the Tyr → Phe Tau mutants were monitored by changes in ThT fluorescence at different time points (graphs show one representative experiment for each mutant). Of the phosphorylated proteins only the 4F/pTyr-394 was able to form fibrils, as detected by the increase in ThT fluorescence over time. Two-way ANOVA with Tukey's multiple comparisons test, significance values are indicated by: *, *p* < 0.05; **, *p* < 0.01; and ***, *p* ≪ 0.001. *C,* at several time points, an aliquot of the aggregation was taken and centrifuged. The supernatants were run on SDS-PAGE. All of the nonphosphorylated mutants showed a significant reduction of the soluble fraction over time. Of the phosphorylated proteins, only the 4F/pTyr-394 presented a similar reduction in the amount of soluble protein over time, whereas the 4F/pTyr-310, pTau-Y310F, and pTau-Y394F formed nonpelletable oligomeric structures (*red dashed rectangles*). *D,* EM micrographs of nonphosphorylated (*top panels*) and phosphorylated (*bottom panels*) Tau Tyr → Phe mutants at 24 h. Of the phosphorylated proteins only the 4F/pY394 was able to form fibrils, whereas 4F/pTyr-310, pTau-Y310F, and pTau-Y394F formed many oligomeric and amorphous structures (*arrows*).

To determine the relative contribution of each tyrosine residue to the c-Abl phosphorylation-mediated inhibition of Tau aggregation, we generated Tau proteins that were phosphorylated or not at one or a selected number of tyrosine residues focusing on Tyr-394 and Tyr-310. To allow site-specific phosphorylation of these residues, we mutated all tyrosine residues except Tyr-310 (4F/Tyr-310) or Tyr-394 (4F/Tyr-394) to phenylalanine. In addition, we generated Tyr-310 and Tyr-394 residue-specific phosphorylation-deficient Tau mutants, where each of these residues is mutated to phenylalanine to allow phosphorylation of all other tyrosine residues except at Tyr-310 (Y310F) or Tyr-394 (Y394F) (Fig. 4*A*, *open circles*). These Tau variants were subjected to *in vitro* phosphorylation using c-Abl (Fig. 4*A*, *red circles*) and the phosphorylated proteins were purified and their aggregation properties were compared with that of the corresponding nonphosphorylated variants. For the 4F/Tyr-310 and 4F/Tyr-394 mutants, which could only be phosphorylated at Tyr-310 and Tyr-394, complete phosphorylation was achieved, whereas a mixture of up to four phosphorylation sites was observed for the single Y310F and Y394F mutants (see LC/MS spectra in Tables 1 and 2). We next investigated the kinetics of aggregation of Tau Tyr → Phe mutants using the same experimental conditions as for full-length Tau (Fig. 4). We observed that 4F/pTyr-310, pTau-Y310F, and pTau-Y394F were unable to aggregate, as demonstrated by the lack of increase in the ThT fluorescence for these samples (Fig. 4*B*), whereas 4F/pTyr-394 retained its capacity to form β-sheet–containing species. These findings were supported by EM data (Fig. 4*D*), where only the 4F/pTyr-394 sample was found to contain Tau of fibrillar morphology, whereas all other phosphorylated mutants only showed the presence of oligomeric species (Fig. 4*D*, *arrows*). These results were further supported by the sedimentation assay, whereupon removal of fibrillar species by ultracentrifugation and quantification of unsedimentable Tau species showed a time-dependent decrease in soluble Tau in all nonphosphorylated mutants (Fig. 4*C*) that paralleled the increase in ThT fluorescence signal (Fig. 4*B*), signifying the recruitment of monomeric Tau into β-sheet–containing species. A time-dependent decrease in monomeric Tau over time was also observed for phosphorylated Tau mutants. However, in contrast to their nonphosphorylated counterparts or mutant 4F/pTyr-394, higher molecular weight Tau bands were apparent for phosphorylated Tau mutants 4F/pTyr-310, pTau-Y310F, and pTau-Y394F (Fig. 4*C*, *red boxes*), suggesting the presence of Tau oligomeric species, which agrees with EM and ThT data. These findings suggest that phosphorylation at Tyr-310, but not Tyr-394 is sufficient to inhibit the fibrillization of Tau, as 4F/pTyr-394 was able to aggregate to the same extent as its nonphosphorylated version. Interestingly, phosphorylation of the Y310F mutant also suppressed Tau fibril formation, even though in this mutant the Tyr-310 residue was not available for phosphorylation, indicating that phosphorylation of multiple N-terminal tyrosine residues (Tyr-18, Tyr-29, and Tyr-197) may also play an important role in inhibiting Tau aggregation. These results show that although Tyr-310 phosphorylation is sufficient to suppress the aggregation of the full-length Tau, the interplay between phosphorylation of the N-terminal tyrosine residues is also of critical importance in modulating Tau oligomerization and fibril formation.

**Table 1:**
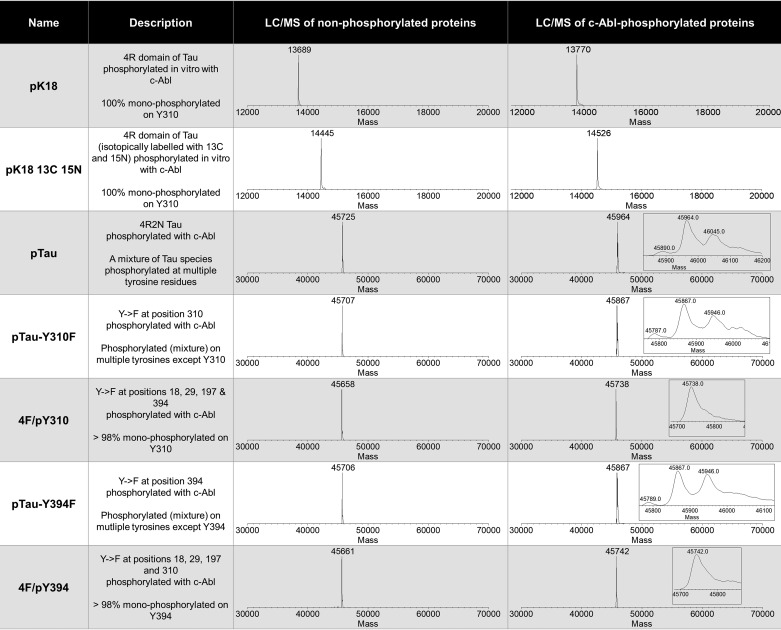
**A list of the phosphorylated proteins used in this study, including how they were prepared and the LC/MS data for each protein before and after *in vitro* phosphorylation with c-Abl**

**Table 2:**
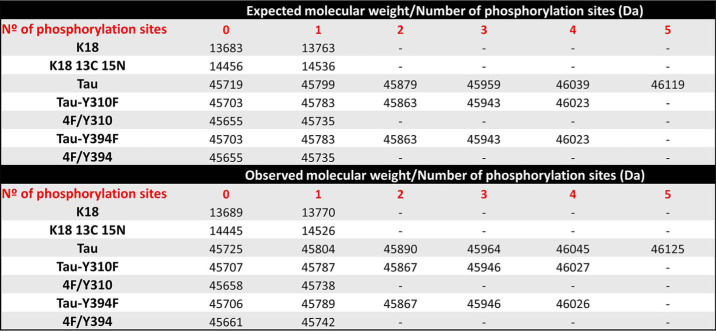
**Expected and observed molecular weights of the proteins used in this study and their phosphorylated states**

### Tyrosine 310 phosphorylation underlies the structural basis of the inhibitory effect on Tau aggregation

The strong inhibitory effects of Tyr-310 phosphorylation combined with the fact that it represents the only tyrosine residue within the MT-binding domain of full-length Tau and the K18 fragment prompted us to investigate the molecular and structural bases by which Tyr-310 phosphorylation inhibits Tau aggregation propensity. Toward this goal, we performed nuclear magnetic resonance (NMR) studies on the K18 peptide, which encompasses the MT-binding region (residues 244–368 of Tau-441 sequence) and includes the highly amyloidogenic PHF6 and PHF6* hexapeptides, the former of which contains Tyr-310 (Fig. 1). K18 is also known for its high propensity to aggregate *in vitro* and in cells (48).

**Figure 5.**
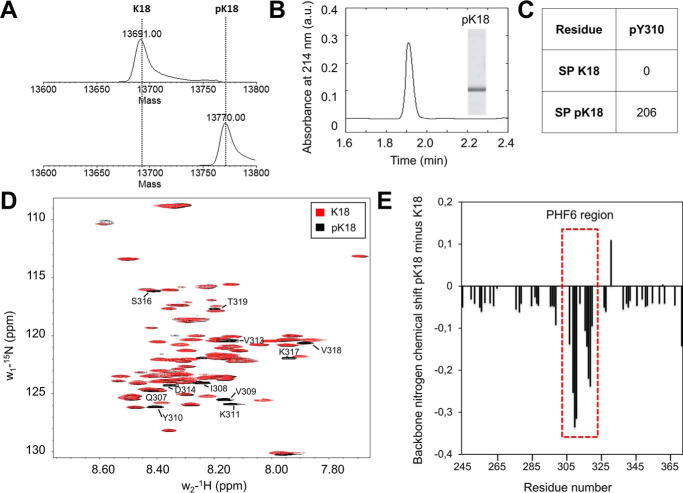
**Detailed characterization of phosphorylated K18 at Tyr-310 (pK18) and NMR analysis of ^15^N pK18 compared with the nonphosphorylated K18.** LC/MS spectra (*A*), UPLC profile and SDS-PAGE (*B*) of reverse-phase HPLC of purified pK18 following phosphorylation by c-Abl (phosphorylation was performed for 4 h). K18 phosphorylated by c-Abl leads to the complete phosphorylation at residue Tyr-310. *C,* the analysis of the phosphopeptides was performed using the Scaffold version 4.0 (Proteome Software), and the number of phosphopeptides detected per phosphorylation site was reported as the SP. *D,* proton-nitrogen HSQC spectrum of unmodified K18 (*red*) compared with that of pK18 (*black*). Phosphorylation on Tyr-310 induced local effects on the structure of pK18, as shown by chemical shift changes to annotated signals. *E,* decreased values of nitrogen chemical shifts (plotted as pK18 minus K18) indicate that phosphorylation decreases the β-sheet propensity of K18 in the PHF6 region, as well as for the subsequent ∼10 residues, all of which are found in a β-sheet conformation in Tau fibrils.

To this end, we produced recombinant ^13^C,^15^N-labeled K18 and subjected it to *in vitro* phosphorylation using recombinant c-Abl under the same experimental conditions as full-length Tau. Given that there is only one tyrosine residue in K18, we were able to achieve complete K18 phosphorylation on Tyr-310 within 4 h and obtained highly pure Tyr-310 specifically modified pK18 (Fig. 5, *A–C*). Next, to determine the effect of Tyr-310 phosphorylation on the structural properties of K18, we performed NMR analysis of ^15^N-labeled pK18. The proton-nitrogen HSQC spectrum of pK18 compared with that of K18 showed that phosphorylation at the Tyr-310 residue induced local effects on the structure of the peptide, as indicated by chemical shift changes for signals originating from residues surrounding Tyr-310 (Fig. 5*D*). Interestingly, signals that were affected in the spectrum of pK18 consistently moved toward lower ^15^N chemical shifts. Because increases/decreases in NMR ^15^N chemical shifts correlate with increases/decreases in local β-strand structure (49), the observed decrease in ^15^N chemical shifts upon Tyr-310 phosphorylation indicated a local decrease in β-sheet propensity for the PHF6 region as well as the subsequent ∼10 residues (Fig. 5*E*). This effect likely results from the added negative charges of the phosphate group. Notably, the affected region adopts β-sheet conformations in all known Tau fibril structures. Thus, the local decrease in β-sheet propensity may have important implications in the context of Tau fibrillization, especially as the PHF6 hexapeptide exhibits the strongest intrinsic β-sheet propensity in the Tau sequence (50) and is critical for the aggregation of the protein (30, 33).

**Figure 6.**
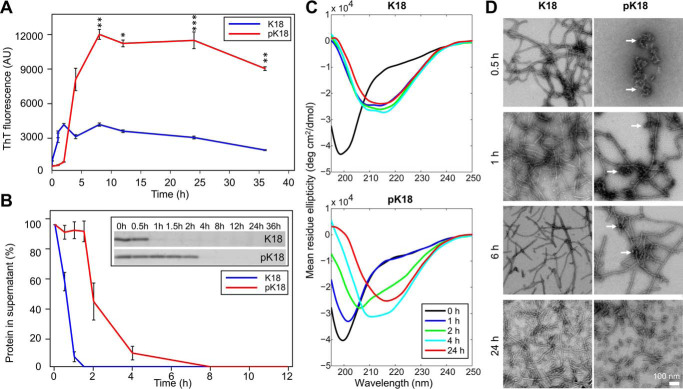
**Comparison of the aggregation behavior of nonphosphorylated and phosphorylated K18.** 10 μm non- and phosphorylated Tyr → Phe Tau mutants were incubated for 48 h at 37 °C under shaking conditions, in the presence of 2.5 μm heparin and the extent of aggregation was monitored by ThT fluorescence (*A*), sedimentation assays (*B*), CD (*C*), and EM (*scale bar* = 100 nm for all images) (*D*). Together, the results from these different assays show that phosphorylation at Tyr-310 has an inhibitory role during the nucleation phase of K18 aggregation, and delays formation of β-sheet–rich K18 fibrils. In *A,* two-way ANOVA with Tukey's multiple comparisons test, significance values are indicated by: *, *p* < 0.05; **, *p* < 0.01; ***, p ≪ 0.001. *B,* at several times points, an aliquot of the aggregation reaction was taken and centrifuged. The supernatants were run on SDS-PAGE (*inset*). The supernatant band intensity from four independent experiments was quantified and reported as the percent of protein remaining in the supernatant (mean ± S.D.).

To further test whether Tyr-310 phosphorylation impacted K18 aggregation, we compared the *in vitro* aggregation of Tyr-310–phosphorylated K18 to that of WT K18 (Fig. 6). As shown in Fig. 6, *A* and *B*, the aggregation of pK18 was significantly delayed compared with unmodified K18. In fact, both the ThT (Fig. 6*A*) and the sedimentation assays (Fig. 6*B*) showed a delay of about 2 h in the fluorescence increase and loss of soluble protein, respectively. Interestingly, the final absolute value of the plateau ThT signal of the phosphorylated K18 was consistently about two to three times higher than that of WT K18, suggesting a difference in fibril structure, packing of the fibrils or susceptibility to fragmentation (Fig. 6*C*), which may affect ThT binding capacity and thus the end-point ThT value.

To further investigate the effect of phosphorylation at Tyr-310 on the early formation of β-sheet–containing species of K18, we monitored changes in K18 secondary structure over time under aggregation conditions by CD spectroscopy. K18 displayed a significantly lower ThT fluorescence signal than pK18 (Fig. 6*A*) but sedimented more rapidly than pK18 (Fig. 6*B*). In addition, K18 fully converted to β-sheet within 1 h, whereas pK18 clearly showed a markedly delayed conformational shift toward β-sheet spectra during 4 h of incubation (Fig. 6*C*). As shown in Fig. 6*D*, EM studies confirmed that fibril formation by pK18 was also delayed and showed significant accumulation of oligomeric amorphous aggregates at early and intermediate time points (Fig. 6*D*, 0.5 h, 1 h, 6 h, *arrows*), and accumulation of fragmented and fully mature fibrillar morphologies, in addition to some oligomers at later time points (Fig. 6*D*, 6 and 24 h). These findings suggest that Tyr-310 phosphorylation results in delayed formation of β-sheet–rich K18 fibrillar structures (Fig. 6, *A* and *B*) and has an inhibitory role during the nucleation phase of K18 aggregation, as demonstrated by attenuation of the pK18 conformational change (Fig. 6*C*).

### Phosphorylation on tyrosine residues reduces Tau affinity for MTs

**Figure 7.**
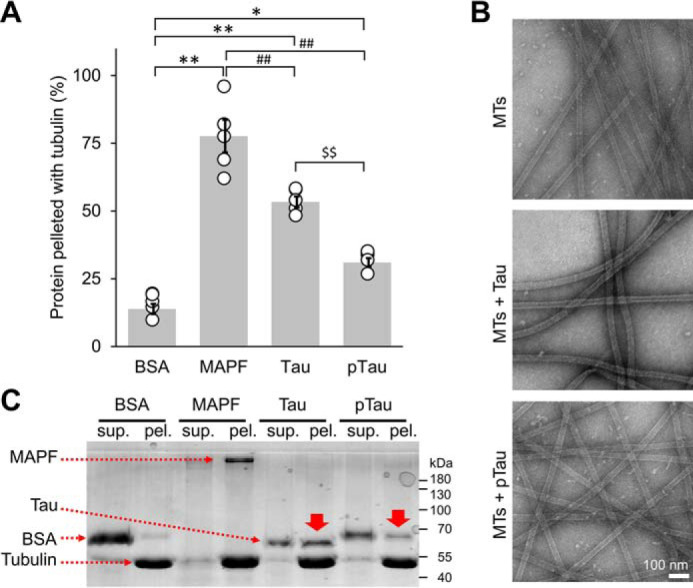
**Tau/microtubule-binding assay.** *A,* the binding propensity of pTau to the MTs was quantified as the percent of protein pelleted when incubated with pre-formed paclitaxel-stabilized MTs at a single concentration (250 μg/ml of pTau and 100 μg/ml of tubulin). Tau bound to tubulin with about 50% efficiency, whereas this efficiency was decreased to about 30% in the case of pTau. As a negative control, we used BSA, which showed low MT binding efficiency of 12%. As a positive control, the MAPF was used and showed a high binding of 78%. The averaged quantification was from 5 repeats, represented as mean ± S.D. with individual points plotted. One-way ANOVA with Tukey's multiple comparisons test was used, significance of values are denoted by: *, *p* < 0.05; **, ## and $$, *p* < 0.001. *B,* EM micrographs of paclitaxel-stabilized MTs alone and incubated with Tau or pTau. *Scale bar* = 100 nm for all images. *C,* representative total protein SDS-PAGE illustrating less pTau protein in the pellet fraction compared with Tau (*solid red arrows*). *sup*. = supernatant; *pel*. = pellet.

In light of the prominent direct association of Tau with MTs, we next asked whether Tau tyrosine phosphorylation, especially on residues Tyr-310 and Tyr-394, influence Tau microtubule-binding propensity. To address this question, we used full-length Tau phosphorylated by c-Abl, taking advantage of the preferential phosphorylation of Tyr-310 and Tyr-394 among other residues, as demonstrated by MS SP counts (Fig. 2*D*). We first assessed the binding propensity of pTau to the MTs by quantifying the percent of protein pelleted when incubated with pre-formed paclitaxel-stabilized MTs at a single concentration (250 μg/ml Tau and 100 μg/ml of tubulin) (Fig. 7*A*). We observed, as previously reported (16), that Tau bound to tubulin with efficiency about 50%, whereas this efficiency was decreased to ∼30% in the case of pTau. As a negative control, the BSA was found to bind MTs with low efficiency of 12%, and as a positive control, the microtubule-associated protein fraction (MAPF) showed a high binding of 78% (Fig. 7*A*). These results were further confirmed by SDS-PAGE, where a band corresponding to the molecular weight of nonphosphorylated Tau was enriched in the pellet fraction (Fig. 7*C*, *solid red arrows*), whereas pTau remained predominantly in the soluble fraction. EM data showed no morphological differences for MTs bound to Tau or pTau (Fig. 7*B*). Our results confirm the negative impact of tyrosine phosphorylation on the interaction of Tau with MTs, likely mediated by the large shift toward the negative electrostatic potential as well as structural rearrangements of the MT-binding region through the phosphorylation of C-terminal Tyr-310 and Tyr-394, with a possible contribution from other phosphorylated tyrosine residues requiring further investigation.

### Phosphorylation on tyrosine residues reduces Tau affinity for and binding of lipid vesicles

We have previously shown that both Tau and K18 interact with brain phosphatidylserine (BPS) vesicles resulting in vesicle disruption and the formation of highly stable oligomeric protein-phospholipid complexes (52). Therefore, we sought to assess whether phosphorylation on tyrosine residues influenced Tau interaction with membranous vesicles and the formation of such Tau-phospholipid complexes.

**Figure 8.**
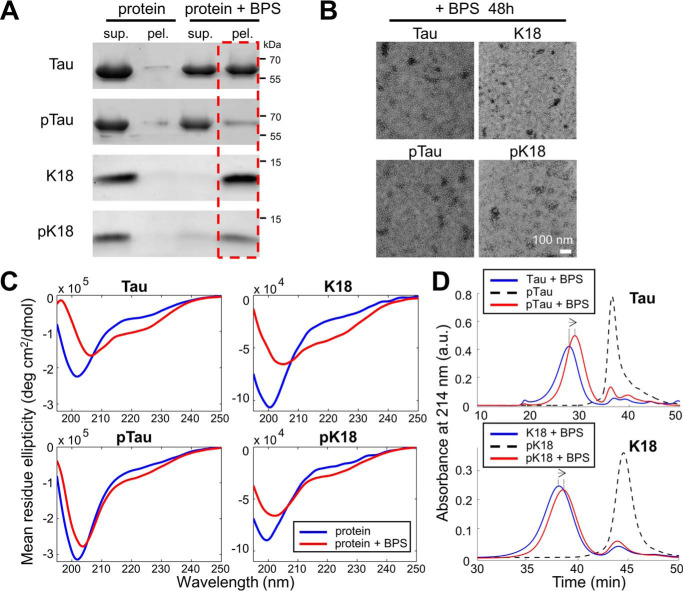
**Phosphorylation on tyrosine residues reduces Tau affinity for and binding of lipid vesicles.** *A,* co-sedimentation assay of BPS vesicles with 10 μm Tau, K18, pTau, and pK18 at a molar ratio 1:20 (protein:phospholipid) incubated for 2 h before analysis. *B,* EM micrographs showing both WT and phosphorylated proteins were able to form phospholipid/protein complexes. *Scale bar* = 100 nm for all images. *C,* CD spectra of proteins incubated for 2 h alone (*blue*) or in the presence of BPS vesicles at a molar protein:phospholipid ratio of 1:20 (*red*). *D,* assessment of the extent of Tau/K18, pTau/pK18-phospholipids complex formation using size-exclusion chromatography under the same conditions used in *A. Dotted lines* demarcate the elution peaks, *arrows* show the direction of the peak shift.

To address this question, we incubated Tau and K18 and their respective tyrosine-phosphorylated forms with BPS vesicles at a molar Tau to phospholipid ratio of 1:20 and performed sedimentation assays. We found that Tau and K18 incubated with BPS vesicles co-sedimented with vesicles to a larger extent compared with their phosphorylated counterparts. This is demonstrated by the reduction of phosphorylated Tau and K18 in the pellet fractions (Fig. 8*A*, *red box*), signifying reduced Tau affinity and binding to lipids.

Next, to confirm the formation of oligomeric Tau- and K18-phospholipid complexes, EM was performed, where oligomeric complexes were detected for all proteins without discernible morphological differences (Fig. 8*B*), indicating no major differences in the absolute capacity of pTau and pK18 to form oligomeric structures compared with their nonphosphorylated counterparts. These findings were further supported by CD spectroscopy (Fig. 8*C*) that showed the conformational changes in proteins due to formation of oligomeric species by both nonphosphorylated and phosphorylated Tau and K18.

Next, to verify the formation of the Tau/K18 phospholipid-oligomer complexes by the nonphosphorylated and phosphorylated Tau and K18, size exclusion chromatography (SEC) was performed. pTau and pK18 complexes peaks (Fig. 8*D*, *red*) systematically eluted later compared with their nonphosphorylated counterparts (Fig. 8*D*, *blue*; *dashed lines* indicate peaks of the elution profiles, *arrows* indicate the direction of elution profile shift). These data are well in line with biochemical data (Fig. 8*A*) and suggest that Tau-lipid complex formation is affected by tyrosine phosphorylation of Tau and K18, resulting in oligomeric Tau- and K18-phospholipid complexes of smaller size, either through the reduction of the number of protein and/or phospholipid molecules per complex, through complex compaction.

Tau and K18 were reported to undergo the structural transitions upon binding to lipid membranes (28, 53–55). Therefore, to investigate the effects of tyrosine phosphorylation on Tau-phospholipids interactions, we monitored changes in Tau and K18 secondary structures by CD spectroscopy. Interestingly, the conformational transitions toward more structured forms of the proteins in the presence of BPS vesicles were markedly more pronounced for nonphosphorylated Tau and K18 (Fig. 8*C*).

Our results demonstrate that phosphorylated Tau and K18 had reduced lipid vesicle binding affinity and formed oligomers that showed less pronounced structural conformation transitions and SEC elution profiles that were shifted toward smaller species compared with nonphosphorylated Tau and K18 in the presence of BPS vesicles. However, the capacity to associate with lipids was still preserved in the phosphorylated variants. These observations combined with the differences in the SEC and CD profiles of phosphorylated Tau and K18 compared with the unmodified proteins suggest that the oligomers formed by the phosphorylated proteins exhibit distinct conformational and size properties.

### Tyrosine 310 phosphorylation attenuates Tau affinity for and binding of lipid vesicles

Next, we sought to determine the underlying structural basis for the reduced lipid-binding affinity of tyrosine-phosphorylated Tau. Previously, we showed that the core of the highly-stable oligomeric Tau-phospholipid complexes was comprised of both the PHF6* and PHF6 hexapeptide motifs, the latter in a β-sheet conformation (52). Because the Tyr-310 residue is located in the PHF6 hexapeptide, part of the R3 repeat peptide (Fig. 1), which plays an important role in mediating the interaction between Tau and membranes (52, 56), we set out to assess the effect of R3 Tyr-310 phosphorylation on the lipid-mediated formation of fibrils by R3 and its interaction with membranes *in vitro*.

**Figure 9.**
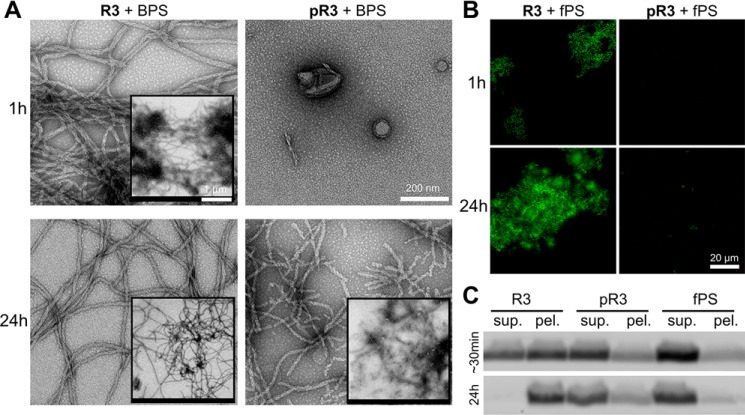
**Tyrosine 310 phosphorylation attenuates R3 peptide affinity for and binding of lipid vesicles.** *A,* EM and *B,* fluorescence microscopy images of 100 μm R3 and pR3 peptides incubated with BPS vesicles at a molar ratio of 1:1. For fluorescence microscopy, R3 or pR3 peptides were incubated with vesicles containing 1% fluorescent NBD-labeled phospholipids (fPS). *Scale bar* in *A* = 200 nm; in *insets* = 1 μm. *Scale bar* in *B* = 20 μm for all images. *C,* SDS-PAGE gel analysis of the fluorescent lipid signal detection of the lipid vesicle flotation and peptide-lipid complex sedimentation assays. ∼30 min sample represents aliquots taken immediately after addition of peptides to fPS vesicles. Presence of a faint signal in the pellet fraction of R3-fPS sample is likely due to fast association of peptide with lipids that occurred during the short period of time required to take and process the sample (estimated time ∼30 min).

First, we incubated R3 and pR3 peptides with BPS vesicles at a molar peptide to phospholipid ratio of 1:1 and monitored fibril formation using EM (Fig. 9*A*). We observed a very rapid formation of large fibrils by R3, whereas the pR3 peptide exhibited a significant delay in fibril formation (Fig. 9*A*, 1 h). Furthermore, whereas R3 formed long fibrils at a later time point, pR3 formed relatively shorter fibrils (Fig. 9*A*, 24 h, *insets*).

Our *in vitro* aggregation studies suggested that the phospholipids become incorporated within the fibrils during the fibrilization of R3. Therefore, we assessed the effects of Tyr-310 phosphorylation on R3 binding and incorporation of phospholipids into R3 fibrills. To this end, we co-incubated the R3 and pR3 peptides with vesicles containing 1% NBD-labeled fluorescent phospholipids (fPS). Under these conditions, we observed the formation of fluorescent fibrils in the case of R3, suggesting that R3 bound tofibrils in the case of R3, suggesting that R3 bound to and incorporated phospholipids within the fibril matrix. No fluorescent signal was observed in the case of the fibrils formed by pR3, suggesting that they were devoid of phospholipids (Fig. 9*B*).

To further confirm the interaction of R3 and pR3 with the phospholipids, we performed a peptide-lipid sedimentation assay based on free lipid vesicle flotation. R3 and pR3 were incubated for 24 h with BPS vesicles containing 1% NBD-labeled fluorescent phospholipids, subsequently centrifuged, and run on SDS-PAGE and imaged with a fluorescent scanner, which detects fluorescent lipid signal (Fig. 9*C*). In this assay, vesicles alone floated and remained in the supernatant (Fig. 9*C*, *fPS, sup*.) and did not pellet to a high extent. However, upon association with the R3 peptide, a fluorescent signal could be observed in the pellet fractions. The signal present in the pellet fraction of R3-fPS at ∼30 min was likely due to the high aggregation propensity of R3 and aggregation that occured during the short of period of time required to aliquot and process the sample. We observed that phospholipids in the R3 sample were solely present in the pellet fraction after 24 h incubation, but that only a small fraction of the phospholipids in the pR3 sample were found in the pellet (Fig. 9*C*). These results further confirm our EM and fluorescence microscopy data and indicate that the capacity of R3 to form fibrils that contain phospholipids in the presence of BPS vesicles was significantly diminished by phosphorylation of residue Tyr-310. These findings are in line with data obtained with the full-length Tau and K18 data (Fig. 8), and further suggest that lipid incorporation into the fibrillar matrix is likely mediated by the aggregation-prone PHF6 peptide.

## Discussion

Despite the presence of tyrosine residues in domains that regulate Tau interactions with microtubules and its aggregation propensity, the role of Tau tyrosine phosphorylation in regulating Tau functions, structure, aggregation, and toxicity remain generally understudied. For example, tyrosine 310 is located in the PHF6 motif of the Tau protein, which has been shown to form a β-sheet structure in the core of Tau fibrils and is necessary for Tau aggregation *in vitro*. Despite consistent findings implicating PHF6 in the initiation of Tau aggregation, stabilization of Tau fibrils, and the importance of the interaction of Tyr-310 and Ile-308 for Tau filament formation (35), there are no reports in the literature examining the potential impact of phosphorylation at this residue on the physiological and/or pathogenic properties of biologically relevant full-length Tau proteoforms. This is possibly due to the lack of well-characterized pTyr-310–specific antibodies and until recently the lack of methods for site-specific phosphorylation of Tau (57–59).

**Figure 10.**
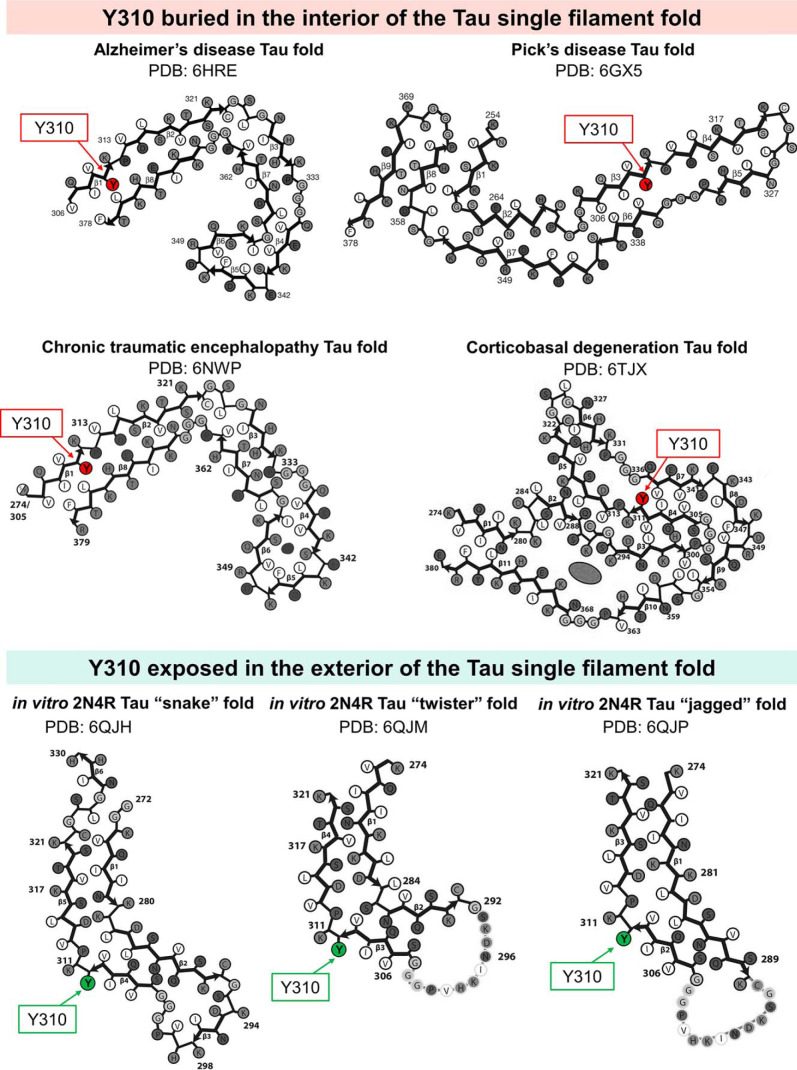
**Schematic representations of Tau filament core-fold from recently published cryo-EM structures of brain-derived and *in vitro* generated Tau filaments.** Tyr-310 residue is buried in the interior (*red*) of the Tau single filament-fold in structures derived from AD, Pick's disease, CTE, and CBD-Tau, or exposed in the exterior (*green*) in *in vitro* derived Tau structures. Adapted from Refs. 29, 51, and 63–65.

We speculated that modifications, such as the addition of bulky negatively-charged phospho-group to Tyr-310 and/or other tyrosine residues would significantly alter the biophysical, aggregation, and MT-binding properties of Tau. In addition, the stoichiometry and localization of Tau tyrosine phosphorylation may confer differential effects on either the N-terminal domain (Tyr-18, Tyr-29), which is implicated in Tau secretion (60) and regulation of microtubule stabilization (61), or on the mid- and C-terminal domains (Tyr-197, Tyr-310, and Tyr-394), which are implicated in the modulation of Tau aggregation, microtubule- and lipid-binding properties (for a recent review, see Ref. 62). Furthermore, recent studies using cryo-EM of Tau filaments from human brain confirmed the PHF6 motif as a core component of Tau filaments, with Tyr-310 buried within the interior of the Tau filament in Pick's disease, as well as in PHFs and SFs in AD, and Tau filaments in CTE (63–65) (Fig. 10). These findings support our initial rationale for initiating the studies outlined in this manuscript. Although the extrapolations of *in vitro* studies' results to *in vivo* conditions have obvious limitations and must be applied cautiously, the phosphorylation of Tyr-310 residue, which is buried inside the core of Tau filaments in some tauopathies, may have implications for some forms of disease featuring specific Tau strains.

Our results indicate that (*a*) phosphorylation of Tau at multiple tyrosine residues inhibits Tau aggregation through cumulative effects of phosphorylation at multiple or all tyrosine residues, and that (*b*) phosphorylation at residue Tyr-310 is a key determinant of Tau aggregation *in vitro*. We show that Tyr-310 phosphorylation appears to be sufficient to significantly delay Tau aggregation *in vitro* and reduces the propensity of the microtubule-binding region of Tau to aggregate through attenuation of the PHF6 hexapeptide propensity to adopt β-sheet–rich structure in the context of K18 (Fig. 5) and potentially full-length Tau.

Tyr-310 is the only tyrosine residue located in the MT-binding region of Tau which suggest a potential role of Tyr-310 in regulating Tau affinity for MTs. Our work here shows that phosphorylation on tyrosine residues affects full-length Tau affinity for MTs, in contrast to previous reports, where Tau phosphorylated by Fyn (16), Arg (19), or c-Abl (18) *in vitro* was found to bind to MTs with an efficiency similar to that of the nonphosphorylated protein. These findings suggest that tyrosine phosphorylation of Tau on either Tyr-18 (*i.e.* by Fyn) or Tyr-394 (by c-Abl or Arg) did not significantly alter its affinity for MTs. Our data point toward residue Tyr-310 as an important regulator of Tau-MTs interactions. One notable difference to previous reports is that our studies relied on the use of pTau proteins that are site-specifically phosphorylated at residues Tyr-310 or Tyr-394 (Fig. 2*D*). To the best of our knowledge, this is the first report demonstrating the role of tyrosine phosphorylation and Tyr-310 in particular in regulating Tau MT-binding domain.

The fact that the MT-binding domain of Tau is also responsible for Tau-lipid interactions also points to the potential role of Tyr-310 in regulating Tau affinity for lipid membranes. We have previously demonstrated that the PHF6 hexapeptide located within R3 interacts with negatively charged vesicles, resulting in the formation of β-strand–rich mixed phospholipid/peptide fibrils (52). In this work, we show that tyrosine phosphorylation, at least at residue Tyr-310, reduced Tau binding to negatively-charged lipid membranes, which may have important implications for Tau functions at the cell plasma membrane. Our findings are especially relevant in light of reports demonstrating that Tau tyrosine phosphorylation occurs in the human brain under both physiological and pathological conditions.

Although aberrant serine and threonine phosphorylation of Tau is well-established in the pathology of AD and other tauopathies (66), the contributions of tyrosine phosphorylation to Tau pathology and Tau-mediated toxicity in these disorders remain poorly understood. Several lines of evidence suggest that Tau tyrosine phosphorylation is an early event in the development of AD (67). In 1991, Wood and Zinmeister (68) used pan anti-phosphotyrosine antibodies to demonstrate an increase of nonspecific tyrosine phosphorylation in AD patient's brain tissue of NFT-bearing neuronal somatodendritic and dystrophic neurite compartments, as well as microglia-resembling cells within neuritic plaques. Similarly, Shapiro *et al.* (69) reported increased nonspecific phosphotyrosine immunoreactivity in the frontal cortex and hippocampus of AD patients. Since then, several reports based on antibody recognition or MS have shown that Tau is phosphorylated on residues Tyr-18, Tyr-197, and Tyr-394 (16, 18, 19, 70) in AD and in fetal brain, whereas phosphorylation at positions Tyr-18 (71) and Tyr-394 (18) have been shown to occur under physiological conditions.

Furthermore, in the P301L transgenic mouse model, Vega *et al.* (70) showed that Tau phosphorylation at residues Tyr-197 and Tyr-394 correlated with the formation of Tau aggregates and occurred concurrently with serine and threonine phosphorylation (detected by PHF1 antibody at residues Ser-396/Ser-404 and by CP13 at residues Ser-202/Thr-205) known to be implicated in AD pathogenesis (70). Moreover, two tyrosine kinases, Fyn and c-Abl, which are known to phosphorylate Tau, have been shown to co-localize with Tau in NFTs (18, 72). Interestingly, treatment with Aβ increased the activity of both kinases and led to a subsequent increase in Tau tyrosine phosphorylation and cell death, which could be prevented by treatment with tyrosine kinase inhibitors (73). In addition, Tau tyrosine phosphorylation may be involved in regulating several cellular activities of Tau linked to signaling events at the cell membrane, in lipid rafts and/or in the neuron growth cone (16, 18). Overall, these findings strongly suggest that Tau tyrosine phosphorylation plays important roles in both the healthy state and in neurodegenerative disease conditions (74), and further studies to investigate the specific role of Tau residue Tyr-310 modifications are warranted.

In conclusion, our findings show that Tau phosphorylation on multiple tyrosine residues may have additive effects on inhibiting Tau fibril formation, and attenuating its MT- and lipid-binding affinity. In addition, our findings underscore the unique role of Tyr-310 phosphorylation in regulating these properties of Tau, which is consistent with its position within the aggregation-prone PHF6 hexapeptide, itself located in the MT-binding domain of the protein. We show that phosphorylation at Tyr-310 is sufficient to significantly delay the fibrillization of full-length Tau and K18, and of the R3 repeat fragment. Our results strongly implicate tyrosine phosphorylation of Tau as a novel target for disease-modifying therapies of proteopathic disorders driven by Tau aggregation. Furthermore, our results underscore the critical importance of developing the new tools to assess the extent of Tau tyrosine phosphorylation in different tauopathies and enable investigations of the role of tyrosine phosphorylation in regulating Tau function in health and disease.

## Experimental procedures

### DNA constructs and mutagenesis

The K18 fragments were synthesized by GeneArt Gene Synthesis (Life Technologies) and cloned into the pT7-7 for bacterial expression. Tau 2N4R in pCMV6-XL5 vectors were obtained from OriGene Technologies, Inc. and cloned in pET-15b for bacterial expression. Single-site mutagenesis was performed in the pET-15b using the QuikChange Site-directed Mutagenesis Kit (Stratagene), as per the manufacturer's instructions. pET21d SH2-CD c-Abl T231R and pCDFDuet-1 YopH for bacterial expression were kindly donated by Prof. Oliver Hantschel (ISREC Institute, EPFL).

### Peptides

The R3 (^306^VQIVYKPVDLSKVTSKCGSLGNIHHK^331^ and the pY-R3 (^306^VQIV(pY)KPVDLSKVTSKCGSLGNIHHK^331^) corresponding to the third microtubule-binding repeat without/with phosphorylation at Tyr-310 were purchased from CS Bio Co.

### Other compounds

Tubulin protein isolated from porcine brain (97 or >99% pure), provided as a lyophilized powder was purchased from Cytoskeleton Inc. Paclitaxel, from the pacific yew tree *Taxus brevifolia* (purity of >99.5%) was used to inhibit microtubule depolymerization, was provided as a lyophilized powder and resuspended at 2 mm anhydrous DMSO purchased from Cytoskeleton Inc. GTP, provided as a lyophilized powder, was resolubilized at 100 mm with ice-cold MilliQ water purchased from Cytoskeleton Inc. MAPF, isolated from bovine brain, was used as positive control for the MT-binding assay, and supplied as lyophilized powder purchased from Cytoskeleton Inc. Phosphatidylserine isolated from porcine brain (BPS) and fluorescently labeled phospholipid 1-oleoyl-2-{6-[(7-nitro-2–1,3-benzoxadiazol-4-yl)amino]hexanoyl-*sn*-glycero-3-phosphoserine (fPS) were purchased from Avanti Polar Lipids, Inc. Heparin sodium salt was from Applichem GmbH.

### Protein expression and purification (Tau and K18)

Tau isoforms 2N4R in pET-15b and K18 in pT7-7 were expressed in *Escherichia coli* strain BL21. The purification protocol of Tau 2N4R Tau was adapted from Ref. 75. Cells were pelleted and broken by sonication in lysis buffer (3 m urea in 10 mm MES, pH 6.5, 1 mm DTT, 1 mm EDTA, 1 mm PMSF). After centrifugation at 150,000 × *g* for 1 h at 4 °C, 1% (w/v) of streptomycin sulfate was added to the supernatant, and the solution was stirred for 90 min at 4 °C. After centrifugation at 27,000 × *g* for 1 h at 4 °C, the supernatant was dialyzed overnight at 4 °C in ion exchange (IEX) buffer A (10 mm MES, pH 6.5, 20 mm NaCl, 1 mm DTT, 1 mm EDTA). The supernatant was filtered and loaded on a cation-exchange column (MonoS, GE Healthcare) and the protein was eluted using a salt gradient (increasing the NaCl concentration of IEX buffer A from 20 mm to 1 m NaCl over 20 column volumes). Fractions containing the proteins were dialyzed overnight against acetic buffer (5% acetic acid in water) and loaded on a reverse-phase HPLC C4 column (PROTO 300 C4, 10 μm, Higgins Analytical; buffer A, 0.1% TFA in water; buffer B; 0.1% TFA in acetonitrile), and the protein was eluted using a gradient from 30 to 40% buffer B over 40 min (15 ml/min). K18 in pT7-7 was purified following a protocol adapted from Ref. 76. Briefly, cells were broken by sonication in IEX buffer B (10 mm HEPES, pH 6.5, 1 mm MgCl_2_, 20 mm NaCl, 1 mm DTT, 1 mm PMSF). After centrifugation at 40,000 × *g* for 30 min, the supernatant was boiled until the solution became cloudy (∼5 min) and the supernatant was centrifuged again for 30 min. The supernatant was filtered and loaded on a cation-exchange column (MonoS, GE Healthcare), and the protein was eluted using a salt gradient (increasing the NaCl concentration of IEX buffer B from 20 mm to 1 m NaCl over 20 column volumes). Fractions containing the K18 fragment were immediately loaded on a reverse-phase HPLC C4 column (PROTO 300 C4 10 μm, Higgins Analytical; buffer A, 0.1% TFA in water; buffer B, 0.1% TFA in acetonitrile), and the protein was eluted using a gradient from 30 to 40% buffer B over 40 min (15 ml/min).

### SH2-CD c-Abl expression and purification

Recombinant SH2-CD c-Abl, T231R, and YopH were co-overexpressed and SH2-CD c-Abl was purified from *E. coli* as previously described (77). Briefly, cells were grown in 2 liters of TB media until the OD reached 1.0, after which the cultures were cooled down and induced overnight at 18 °C with 0.2 mm isopropyl 1-thio-β-d-galactopyranoside. The cells were then lysed by sonication in His-tag binding buffer HA (50 mm Tris, pH 8, 500 mm NaCl, 5% glycerol, 25 mm imidazole) and centrifuged twice at high speed (50,000 × *g*, 20 min, 4 °C), before being injected in 5 ml of His-tag columns (histrap 5-ml column, GE Healthcare; buffer HA, same as above; buffer HB, same as HA with 0.5 m imidazole). Selected fractions were desalted using two HiPrep 26/10 desalting columns (GE Healthcare) in series (desalting buffer: 20 mm Tris, pH 8, 50 mm NaCl, 5% glycerol, 1 mm DTT), combined and further purified by anion-exchange chromatography using a MonoQ 5/50 GL column (GE Healthcare; buffer A, 20 mm Tris, 5% glycerol, 1 mm DTT, pH 8.0; buffer B, same as buffer A with 1 m NaCl). The final concentration of the c-Abl kinase was determined using the UV absorbance at 280 nm (m = 46797 g mol^−1^ and ϵ_280_ = 80,010 m^−1^ cm^−1^).

### Large-scale preparation of phosphorylated K18, Tau, and Tyr → Phe mutants by c-Abl

The large-scale phosphorylation of 10 mg of K18, Tau, and Tau Tyr → Phe mutants were performed for 4 h in 50 mm Tris, 5 mm MgCl_2_, 1 mm DTT, 20 mm Na_3_VO_4_ (phosphatase inhibitor) in the presence of 3 mm MgATP, pH 7.5, at 30 °C. We used c-Abl kinase at a mass to mass ratio of 1:20 (kinase:Tau). The reaction mixture was followed by ESI-MS to verify the completion of the phosphorylation. Additional kinase and MgATP were added when needed. Phosphorylated K18, Tau, and Tyr → Phe mutants were purified by a reverse-phase HPLC preparative C4 column (PROTO 300 C4 10 μm Higgins Analytical; buffer A, 0.1% TFA in water; buffer B, 0.1% TFA in acetonitrile) using a linear gradient of 30 to 40% of B in 40 min for Tau or 20 to 35% of B in 40 min for K18. The HPLC fractions were analyzed by LC-MS and once pure were pooled and lyophilized.

### Preparation and characterization of heparin-induced Tau and K18 fibrils

Fibrils of WT, mutants, and phosphorylated Tau and K18 were formed by incubating monomeric protein in 10 mm phosphate, pH 7.4, 50 mm NaF, and 0.5 mm DTT with heparin sodium salt (Applichem GmbH, activity 204.7 IU/mg; molecular weight 8,000–25,000 g/mol) at a molar heparin:protein ratio of 1:4 under constant orbital agitation (1000 rpm, Peqlab, Thriller) at 37 °C for up to 72 h.

### R3 and pY-R3 peptide aggregation

100 μm R3 or pY-R3 peptide were dissolved in 10 mm Tris, 50 mm NaF, 0.5 mm DTT and pH adjusted to 7.4. The solution was then vortexed, centrifuged for 20 min at 14,000 rpm, and the supernatant was incubated in the absence or presence of 25 μm heparin sodium salt (Applichem GmbH) at 37 °C, with constant orbital agitation (1000 rpm, Peqlab, Thriller).

### Vesicle preparations

Vesicles were prepared using the extrusion method. Briefly, individual phospholipids or phospholipid mixtures in chloroform were dried using an argon stream to form a thin film on the wall of a glass vial. Potentially remaining chloroform was removed by placing the vial under vacuum overnight. The phospholipids were resolubilized in 10 mm HEPES, pH 7.4, 100 mm NaCl, and 2.5 mm DTT to the desired final concentration by sonication. The solution was then passed through an Avestin LiposoFast extruder (Avestin Inc.) (membrane pore size: 0.1 μm), and the size and homogeneity of the resulting vesicles were assessed by EM. For the preparation of fluorescently labeled vesicles, fluorescent and nonfluorescent phospholipids in chloroform were mixed at a molar ratio of 1:99 prior to the initial drying step.

### Preparation of mixed protein-phospholipid complexes

Protein-phospholipid complexes were prepared by incubating BPS vesicles with Tau or K18 at a molar protein:phospholipid ratio of 1:20 in 10 mm HEPES, pH 7.4, 100 mm NaCl, 2.5 mm DTT for 48 h at 37 °C. The resulting protein-phospholipid complexes were separated from the remaining vesicles and soluble protein by SEC using a Superose 6 column (GE Healthcare).

### Preparation of mixed R3 and pY-R3 peptides/phospholipid fibrils

100 μm R3 and pY-R3 peptides were incubated with BPS vesicles at a molar ratio of 1:1. For fluorescence microscopy, the peptides were incubated with vesicles containing 1% fluorescent NBD-phospholipids, imaged with confocal laser-scanning microscope (LSM 700, Zeiss), and analyzed using Zen software.

### Preparation of microtubules

Tubulin was dissolved at a concentration of 5 mg/ml in ice-cold General tubulin buffer (80 mm PIPES, 0.5 mm EGTA, 2 mm MgCl_2_, pH 6.9) supplemented with 1 mm GTP and snap-frozen as 10-μl aliquots in liquid nitrogen and stored at −80 °C. Microtubules were assembled by defrosting an aliquot of 10 μl of tubulin stock in a room temperature water bath, immediately placed on ice, and incubated for 20 min at 35 °C in 100 μl of pre-warmed (35 °C) General tubulin buffer supplemented with 1 mm GTP and 5% glycerol. Then the solution was diluted with 100 μl of General tubulin buffer supplemented with 1 μl of paclitaxel (Taxol,® stock at 2 mm), gently mixed, and kept at room temperature. Microtubule morphology was systematically verified by EM.

### Microtubule-binding assay

Pre-formed paclitaxel-stabilized MTs were incubated for 30 min at room temperature with Tau, pTau, MAPF, or bovine serum albumin (BSA) (250 μg/ml of protein and 100 μg/ml of tubulin) in General tubulin buffer supplemented with 1 mm GTP and 5% glycerol (total of 50 μl per sample). The samples were placed on top of a 100 μl of cushion buffer (*i.e.* General tubulin buffer containing 60% glycerol) in 1.5-ml tubes. The tubes were centrifuged at 100,000 × *g* for 40 min at room temperature. The top 50 μl of each tube was mixed with 10 μl of 5× Laemmli buffer (supernatant) and the pellet was resuspended in 50 μl of 1× Laemmli buffer (pellet). The samples were run on SDS-PAGE gels. The relative amounts of Tau, pTau, MAPF, or BSA in the supernatant and in the pellet were estimated by measuring the band intensity using Fiji software (National Institute of Health) over 5 repeats, and represented as mean ± S.D.

### Monitoring of c-Abl Tau/K18 phosphorylation by MS

100 or 200 μg of WT Tau or K18 were incubated with 5 or 10 μg of recombinant c-Abl in 50 mm Tris, 5 mm MgCl_2_, 1 mm DTT, 20 mm Na_3_VO_4_ (phosphatase inhibitor), 3 mm MgATP, pH 7.5. Reactions were performed for 4 h at 30 °C without agitation. The reactions were followed using ESI-MS on a Thermo LTQ ion trap system.

### Tandem MS/MS of in vitro c-Abl–phosphorylated recombinant Tau

Tau phosphorylated with c-Abl as described above was run on SDS-PAGE and stained using Simply Blue Safe Stain (Life Technologies). The bands corresponding to Tau were excised, destained with 50% ethanol in 100 mm ammonium bicarbonate, pH 8.4, and dried. The proteins were digested in gel by covering gel pieces with a trypsin solution (12.5 ng/μl in 50 mm ammonium bicarbonate, pH 8.4, 10 mm calcium chloride) overnight at 37 °C. The digested peptides were subjected to TiO_2_ phosphopeptide enrichment, concentrated to dryness, resuspended in acetonitrile, and dried again. After repeating this step, the dried peptides were resuspended in 20 μl of 20% formic acid, and 2% acetonitrile, desalted on C18 OMIX tips (Agilent Technologies), and analyzed by a capillary LC-ESI-MS system (Thermo Scientific LTQ Orbitrap instrument). The collected MS/MS spectra were matched against human and scored using Mascot and Sequest algorithms. The data were then analyzed using Scaffold version 4.0 (Proteome Software, (RRID:SCR_01435).

### Transmission EM

Samples (3.5 μl) were applied onto glow-discharged Formvar/carbon-coated 200-mesh copper grids (Electron Microscopy Sciences) for 1 min. The grids were blotted with filter paper, washed twice with ultrapure water and once with staining solution (uranyl formate, 0.7% (w/v)), and then stained with uranyl formate for 30 s. The grids were blotted and dried. Specimens were inspected with a Tecnai Spirit BioTWIN operated at 80 kV and equipped with an LaB6 filament and a 4K × 4K FEI Eagle CCD camera.

### Circular dichroism (CD) spectroscopy

CD spectra were recorded on a Jasco J-815 CD spectrometer operated at 20 °C. To minimize buffer absorption, the buffer was modified to 10 mm phosphate buffer, pH 7.4, with 50 mm NaF and 0.5 mm DTT. CD spectra were acquired from 195 to 250 nm in 0.2-nm increments at a 50 nm/min scan rate. For each sample, five spectra were averaged and smoothed using binomial approximation.

### NMR spectroscopy

^1^H-^15^N HSQC spectra were collected at 25 °C on a Bruker Avance 600 MHz spectrometer using samples containing 500 μm K18 or pK18 peptide in 10 mm Na_2_HPO_4_, 100 mm NaCl, 1 mm DTT, 10% D_2_O buffer at pH 7.4. Resonance assignments are based on the previously published assignments of the K18 spectrum (66).

### Sedimentation assay

Taking advantage of the fact that high-speed centrifugation sediments fibrils but not monomeric or soluble oligomeric species, the sedimentation assay makes it possible to assess the ratio of fibrillized to soluble protein. 20 μl of WT Tau, K18, or Tyr → Phe mutants aggregation reactions were pelleted by ultracentrifugation for 2 h at 200,000 × *g* at 4 °C. The supernatant was mixed with 2× Laemmli buffer and run on 15% SDS-PAGE. When stated, the amount of soluble material was quantified by measuring the band intensity using Fiji software (National Institute of Health) and averaged over at least three independent repeats.

### Thioflavin T fluorescence measurements

Thioflavin T (ThT) fluorescence reading (excitation wavelength of 450 nm, emission wavelength of 485 nm) was performed in triplicate with a ThT concentration of 60 μm and peptide concentration of 60 μm or a protein concentration of 3 μm in 50 mm glycine, pH 8.5, using a Bucher Analyst AD plate reader without shaking.

### Vesicle-binding assay

The co-sedimentation assay was used to assess initial binding of protein to vesicles. Tau or K18 WT, or tyrosine phosphorylated (10 μm) were mixed with BPS vesicles (200 μm) to a total volume of 20 μl and immediately centrifuged for 2 h at 200,000 × *g* at 4 °C. At this speed, the vesicles pellet, and protein bound to them co-sediment with the vesicles. Both supernatant, containing unbound protein, and the resuspended pellet, containing vesicles with bound protein, were run on 15% SDS-PAGE gels.

## Data availability

All data are provided in manuscript and raw files are available upon request from the corresponding author.

## Supplementary Material

Supporting Information
