# Supplementary material for: Phosphorylation of the overlooked tyrosine 310 regulates the structure, aggregation, and microtubule- and lipid-binding properties of Tau
Source: J Biol Chem. 2020 Apr 27;295(23):7905–22. doi: 10.1074/jbc.RA119.012517 (PMC7278352; doi:10.1074/jbc.RA119.012517)
Supplement: Supporting Information [file supp_295_23_7905__index.html]

Phosphorylation of the overlooked tyrosine 310 regulates the structure, aggregation, and microtubule- and lipid-binding properties of Tau — Phosphorylation at Y310 regulates Tau biophysical properties — Phosphorylation of the overlooked tyrosine 310 regulates the structure, aggregation, and microtubule- and lipid-binding properties of Tau — Phosphorylation at Tyr-310 regulates Tau biophysical properties — Supporting Information 

# Phosphorylation of the overlooked tyrosine 310 regulates the structure, aggregation, and microtubule- and lipid-binding properties of Tau

## Supporting Information

- Supporting Information (to be published online) - SI containing Figure S1 and Legend
