# Supplementary material for: Phosphorylation of the overlooked tyrosine 310 regulates the structure, aggregation, and microtubule- and lipid-binding properties of Tau
Source: J Biol Chem. 2020 Apr 27;295(23):7905–22. doi: 10.1074/jbc.RA119.012517 (PMC7278352; doi:10.1074/jbc.RA119.012517)
Supplement: Supporting Information [file supp_RA119.012517_158022_3_supp_515897_q96kgg.pdf]

Supporting Information

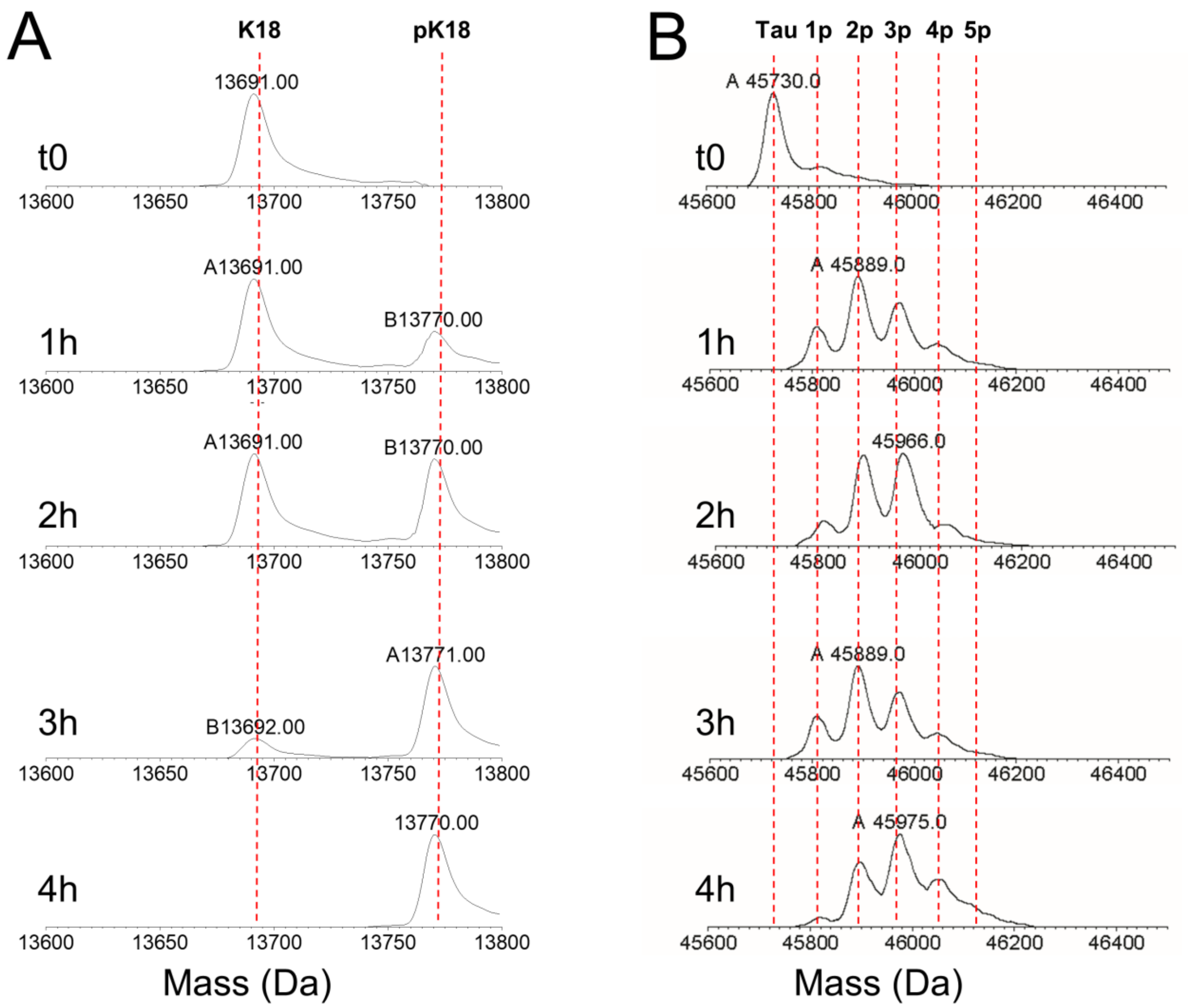

Figure S1. Overtime monitoring of K18 (A) and Tau (B) phosphorylation by c-Abl by LC/MS.
